# Warfarin and coumarin-like *Murraya paniculata* extract down-regulate EpCAM-mediated cell adhesion: individual components versus mixture for studying botanical metastatic chemopreventives

**DOI:** 10.1038/srep30549

**Published:** 2016-08-02

**Authors:** Jingwei Shao, Suxia Zhou, Zhou Jiang, Ting Chi, Ji Ma, Minliang Kuo, Alan Yueh-Luen Lee, Lee Jia

**Affiliations:** 1 Cancer Metastasis Alert and Prevention Center, and Biopharmaceutical Photocatalysis, State Key Laboratory of Photocatalysis on Energy and Environment, Fuzhou University, Fuzhou 350002, China; 2 Fujian Provincial Key Laboratory of Cancer Metastasis Chemoprevention, Fuzhou University, Fuzhou, China; 3 Graduate Institute of Biomedical Sciences, College of Life Science, National Taiwan University, Taipei, Taiwan; 4 National Institute of Cancer Research, 35 Keyan Road, Zhunan, Miaoli 35053, Taiwan

## Abstract

We recently defined cancer metastatic chemoprevention as utilizing safe and effective molecules to comprehensively prevent the spark of activation-adhesion-extravasation-proliferation metastatic cascade caused by circulating tumor cells (CTCs). The strategy focuses on preventing the most important starting point of the cascade. We identified an extract from a well-known medical plant *Murraya paniculata*, which inhibited both embryonic implantation to human endometrium as traditionally-used for abortion and CTC adhesion to human endothelium. Here, we separated and characterized five coumarin-containing components (Z1–Z5) from the botanic extract. Flow cytometry revealed that within 1–100 μg/mL, Z3 and Z5 down-regulated EpCAM expression in human colon HCT116, whereas, Z1 and Z2 did oppositely. Warfarin and Z1-Z5 component mixture (CM) also down-regulated EpCAM expression. The down-regulation of EpCAM by Z3, Z5, CM and warfarin was confirmed by western blotting, and caused inhibition on adhesion of cancer cells to human endothelial cells. Rat coagulation study showed that warfarin prolonged prothrombin time, whereas, Z3 did not. The present studies revealed that, for the first time, warfarin and coumarin-like components Z3, Z5 and CM from *Murraya paniculata* could directly inhibit EpCAM-mediated cell-cell adhesion.

Cancer metastatic spread is a complex process initiated by the activation, dissemination, seeding and engraftment of circulating tumor cells (CTCs). The series of consequential events include the activation of dormant CTCs, and adhesion between CTCs and vascular endothelial bed of metastatic tissues, and the continued survival and initial proliferation of CTCs after extravasation^1^,^2^. We proposed that activation/adhesion of CTCs to the vascular bed is a crucial starting point of the metastatic cascade for chemo-intervention^3^, and if we can control the starting point, we may effectively prevent cancer metastasis.

CTCs are present at low concentrations in the peripheral lymph and blood system of cancer patients and cancer survivors. Many CTCs enrichment and detection methods have been developed, including FISH, immunocytochemistry, RT-PCR, CellSearch system based on the antibody to epithelial cell adhesion molecule (EpCAM; a commonly expressed epithelial cell surface marker^4^). We recently developed a method of immunomagnetic enrichment coupled with flow cytometry to detect CTCs^5^, and characterized colorectal CTCs as EpCAM + CD45-pancytokeratin + from the blood of 18 patients.

Inspired by the CTC characterization study, we targeted the CTC surface biomarker, in particular the EpCAM, by coating nanomaterial dendrimers with EpCAM antibody to specifically capture blood CTCs and restrain their activity without any cytotoxic effects^6^,^7^,^8^. We also demonstrated that a nitric oxide donor compound could inhibit CTCs-initiated metastasis cascade by directly producing vasorelaxation and interfering with hetero-adhesion of cancer cells to vascular endothelium via down-regulating expression of cell adhesion molecules^9^. Very recently, we showed that the highly active metastasis prevention therapy (abbreviated as HAMPT, a combination of four old but safe drugs) can effectively prevent cancer metastasis by acting on intervening inflammatory factors, cell adhesion molecules, selectins, integrins, and platelets to prevent CTCs from seeding on extracellular matrices, and strengthening the extracellular matrices^3^.

Recently we reviewed the current literature and analyzed the molecular and cellular similarities and differences between embryonic implantation to uterine endometrium and CTCs adhesion to vascular endothelium, and found that many molecules, including epithelial cell adhesion molecule (EpCAM), intercellular adhesion molecule (ICAM), vascular cell adhesion molecule (VCAM), selectin, integrin, hormones, sialyl lewis X, and mitochondrial membrane potential (MMP), are shared by both the embryonic implantation and cancer cell adhesion-invasion systems^10^,^11^,^12^,^13^. The analysis led us to screening the traditional abortion Chinese medicinal plants or herbs for potential safe and effective metastatic chemopreventives. In the huge treasure of the Traditional Chinese Medicine (TCM), we hunted for those TCM that should possess the following properties: safe, anti-adhesion, anti-inflammation, anti-coagulation, analgesic, and vasodilation. The TCM *Murraya paniculata* (L.) Jack meets the above criteria^14^,^15^,^16^. The extract from this TCM appeared to be very safe with the oral LD_50_ value to mice > 5 g/kg (the maximum mouse intragastric administration volume)^17^. We first identified the most-promising extracted fraction G (containing flavonoids and coumarins) from the TCM’s raw ethanol/dichloromethane extracts by using the bioactivity-guided fast screen assay^18^. The G extract showed specific inhibition on both embryonic implantation to human endometrial bed and adhesion of cancer cells to human endothelium in a concentration-dependent manner without significant cytotoxicity^19^. The inhibition resulted from down-regulation by the G extract on expression of integrin, CD44, and E-selectin. The extract G also inhibited invasion and migration of cancer cells. Oral administration of the extract G to the immunocompetent mice inoculated with mouse melanoma cells produced significant inhibition on lung metastasis without marked side effects. To continue the previous discovery, in the present study, we used the phytochemically separated components from the root of *Murraya paniculata* (L.) to explore their molecular and cellular bioactivities in preventing adhesion of cancer cells to vascular endothelium after phytochemically characterizing the components (Fig. 1). Since TCM or botanical medicine is often used as an extract that contains various agonistic and antagonistic components (or Ying and Yang components defined by the TCM phrase), and the extract only exhibits a leading pharmacological effect after harmonizing each individual effects, we therefore dissected the anti-adhesion effects of both component mixture (or reconstituting each components together according to their original % proportion in the extract) and individual components by running comparative bioassays in parallel. The studies were reported below.

## Results

### Separation and chemical characterization of individual components from *M. paniculata*

**Figure 2:**
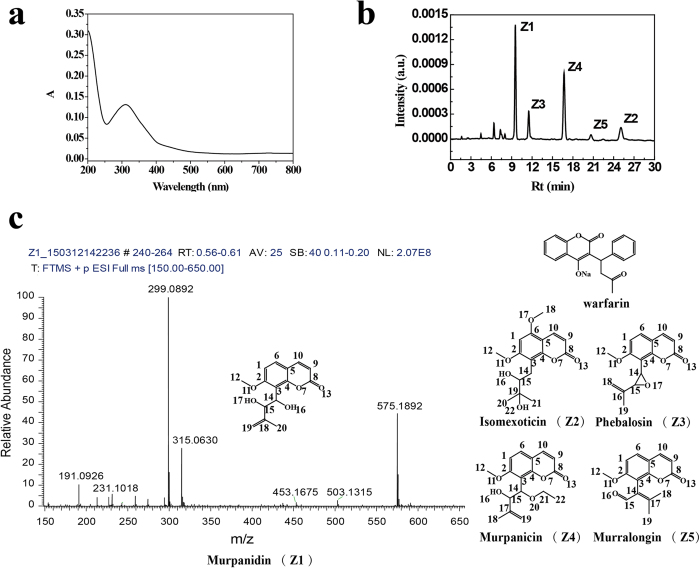
Structure analysis of main components separated from *Murraya paniculata* extract. (**a**) UV-Visible spectroscopic scanning of the extract showing absorption maxima at 310 nm; (**b**) HPLC analytical profile showing five major components existing in the extract, namely, Z1–Z5; (**c**) Mass analysis indicated the m/z values of the five components, which combined with other information on structural analysis (supplementary information) revealed the five components containing coumarin-like structure (i.e., warfarin).

The root of *M. paniculata* (about 15 g) was extracted with 80% refluxing acidic ethanol overnight. The resultant residue was extracted with ethyl acetate as we described previously^18^, which gave about 900 mg of extract mixture (yield 6% w/w). The extract was then dissolved in methanol. The mixture showed high fluorescent intensity, suggesting existence of coumarin-like components that usually exhibit high fluorescence. Separation of individual components was carried out on the HPLC C18 column with isocratic elution of methanol-water (45: 55, v:- v), which gave five components that all showed an absorption maximum at 310 nm (Fig. 2a,b). We collected the five components in amorphous powders after rotary evaporation and lyophilization.

The five major active components were named as Z1 (retention time on 9.53 min), Z2 (25.07 min), Z3 (11.50 min), Z4 (16.70 min), and Z5 (20.67 min) with the peak area ratio of 1.84: 0.817: 1.00: 1.82: 0.213, successively (Fig. 2b). To obtain enough amount of Z1-Z5 for the *in vitro* and *in vivo* assays, we used the semi-preparative HPLC column for separation of the components followed by rotary evaporation and lyophilization. The procedure gave the yield of component Z1 0.50%, Z2 0.068%, Z3 0.19%, Z4 0.34%, and Z5 0.06% (w/w), respectively. After purification, the molecular formula of each component was analyzed and defined by the molecular ion peaks in the positive ion ESI-mass spectrometric mode, and the representative mass spectra were shown in Fig. 2c. The chemical structures of the components were analyzed by using ^1^H NMR and ^13^C NMR spectra. Below are ^1^H NMR and ^13^C NMR data, and based on the data, we assigned Z1 as murpanidin (C_15_H_16_O_5_), Z2, isomexoticin (C_16_H_20_O_6_), Z3, phebalosin (C_15_H_14_O_4_), Z4, murpanicin (C_17_H_20_O_5_), and Z5, murralongin (C_15_H_14_O_4_), respectively^20^,^21^ (Fig. 2). The assignments of each H and C were showed as Supplementary Table S1 and S2.

Z1, ^1^H NMR (500 MHz, DMSO-d_6_): δ 7.96 (dd, J = 9.4, 3.2 Hz, 1 H), 7.67–7.54 (m, 1H), 7.05 (d, J = 8.6 Hz, 1H), 6.28 (dd, J = 9.4, 3.1 Hz, 1H), 5.12 (s, 1H), 5.00 (d, J = 3.5 Hz, 2H), 4.70 (d, J = 8.7 Hz, 1H), 4.50 (s, 1H), 4.42 (s, 1H), 3.88 (d, J = 3.0 Hz, 3H), 1.53 (s, 3H). ^13^C NMR (125 MHz, DMSO-d_6_): δ 160.8, 160.5, 153.1, 146.2, 145.3, 129.2, 117.7, 113.1, 112.6, 112.2, 109, 77.3, 68.2, 56.7, 17.48. ESI-MS: observed 299.0892 [M + Na]^+^, and calculated 299.09 [M + Na]^+^, C_15_H_16_NaO_5_^+^.

Z2, ^1^H NMR (500 MHz, DMSO-d_6_): δ 8.00 (s, 1 H), 6.65 (s, 1 H), 6.15 (s, 1 H), 4.11 (s, 2 H), 3.95 (s, 6 H), 3.46 (s, 1 H), 2.77 (s, 2 H), 1.25 (s, 1 H), 1.13 (s, 6 H). ^13^C NMR (125 MHz, DMSO-d_6_): δ 162.03, 161.01, 155.51, 154.17, 139.46, 110.58, 108.56, 103.18, 92.06, 76.83, 72.39, 56.74, 56.48, 26.05, 25.09, 25.09. ESI-MS: observed 331.1156 [M + Na]^+^, calculated 331.12 [M + Na]^+^, C_16_H_20_NaO_6_^+^.

Z3, ^1^H NMR (500 MHz, DMSO-d_6_): δ 7.97 (d, J = 9.6 Hz, 1 H), 7.60 (d, J = 8.5 Hz, 1 H), 7.07 (d, J = 8.7 Hz, 1 H), 6.28 (d, J = 9.4 Hz, 1 H), 5.07 (s, 1 H), 4.91 (dd, J = 44.7, 36.2 Hz, 1 H), 4.51 (s, 1 H), 4.39 (s, 1 H), 3.88 (s, 3 H), 1.48 (s, 3 H). ^13^C NMR (125 MHz, DMSO-d_6_): δ 162.1, 160.66, 154.25, 147.7, 145.3, 129.41, 115.64, 115.6, 112.74, 112.74, 109, 74.47, 57, 56.49, 19.03. ESI-MS: observed 281.0788 [M + Na]^+^, calculated 281.08 [M + Na]^+^, C_15_H_14_NaO_4_^+^.

Z4, ^1^H NMR (500 MHz, DMSO-d_6_): δ 7.97 (d, J = 9.5 Hz, 1 H), 7.60 (d, J = 8.6 Hz, 1 H), 7.07 (d, J = 8.8 Hz, 1 H), 6.28 (d, J = 9.4 Hz, 1 H), 5.07 (s, 1 H), 4.93 (t, J = 12.5 Hz, 1 H), 4.87 (t, J = 12.5 Hz, 1 H), 4.51(m, 1 H), 4.39 (m, 1 H), 3.88 (s, 3 H), 3.43 (s, 2 H), 1.48 (s, 3 H), 1.06 (t, J = 7.0 Hz, 3 H). ^13^C NMR (125 MHz, DMSO-d_6_): δ161.2, 160.5, 153.5, 145.76, 145.3, 129.8, 114.9, 113.1, 112.7, 112.58, 108.9, 76.55, 76.39, 65.11, 56.85, 17.19. ESI-MS: observed 327.1205 [M + Na]^+^, calculated 327.12 [M + Na]^+^, C_17_H_20_NaO_5_^+^.

Z5, ^1^H NMR (500 MHz, DMSO-d_6_): δ 10.19 (s, 1 H), 8.04 (d, J = 9.5 Hz, 1 H), 7.71 (d, J = 8.7 Hz, 1 H), 7.14 (d, J = 8.7 Hz, 1 H), 6.28 (d, J = 9.5 Hz, 1 H), 3.88 (s, 3 H), 2.41 (s, 3 H), 1.71 (s, 3 H). ^13^C NMR (125 MHz, DMSO-d_6_): δ189.98, 160.67, 160.27,152.48, 145.17, 145.17, 129.65, 129.22, 117.66, 112.59, 112.27, 108.66, 56.7, 24.91, 19.87. ESI-MS: observed 281.0788 [M + Na]^+^, calculated for 281.08 [M + Na]^+^, C_15_H_14_NaO_4_^+^.

### Comparison in cellular and molecular bioactivity between individual components and their mixture

Traditional botanical medicine is usually used as a raw material containing different Ying-Yang components that together produce a combined pharmacological effect on body^22^. Since the five components were extracted together from the root of *M. paniculata* with ethyl acetate, we wanted to compare bioactivity of individual components with their mixture (CM), the latter was obtained before physicochemical separation of individual components, or quantitatively reconstituted by adding the given amount of Z1-Z5 together based on their original proportion existed in the ethyl acetate extract. We used the representative coumarin, i.e., warfarin, as the positive control because the five components contain coumarin structure.

**Figure 3:**
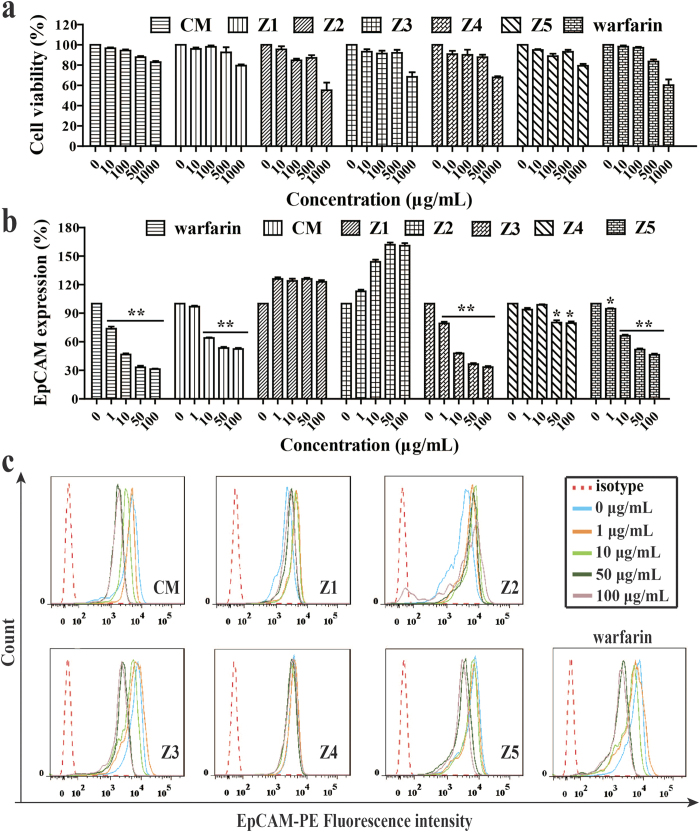
Low cytotoxicity of five components and their effects on adhesion molecule EpCAM of colon cancer cell line HCT116. (**a)** MTT assay showed low cytotoxicity of the five components (Z1~Z5), their mixture (CM) and warfarin against HCT116 when their concentrations reached 500 μg/mL and beyond. (**b)** quantitative flow cytometric analysis revealed concentration-dependent effects of Z1~Z5, CM and warfarin (<100 μg/mL) on expression of EpCAM on HCT116; the results demonstrated that warfarin, CM, Z3, Z4, and Z5 down-regulated EpCAM expression, while Z1 and Z2 did oppositely. (**c**) flow cytometric scanning of effects of warfarin, CM and Z1-Z5 on EpCAM expression using the isotype as the control. Bars represent the mean ± SEM (n = 3). *P < 0.05, and **P < 0.01, compared with the control.

Warfarin, CM and Z1~Z5 showed inappreciable inhibition on HCT116 cells up to 500 μg/mL after 24 h treatment (Fig. 3a). We estimated that their IC_50_ values (the mean drug concentration causing 50% relative growth inhibition of the cells) were all above 1000 μg/mL. Interestedly enough, although warfarin, CM, and Z1-Z5 did not produce any effect on cell viability at concentrations <100 μg/mL, they significantly regulated expression of cellular adhesion molecule EpCAM (Fig. 3b). Among them, Z2 significantly up-regulated the EpCAM expression, followed by Z1. Whereas, Z3, Z4 and Z5 down-regulated the EpCAM expression in a concentration-dependent manner. As a result, CM inclusively produced down-regulation on EpCAM expression. Warfarin also down-regulated EpCAM expression. Figure 3c showed the representative scanning of flow cytometric results, which matched the quantitative analysis (Fig. 3b).

### Effect of CM, Z3 and Z5 on cell-cell adhesion

**Figure 4:**
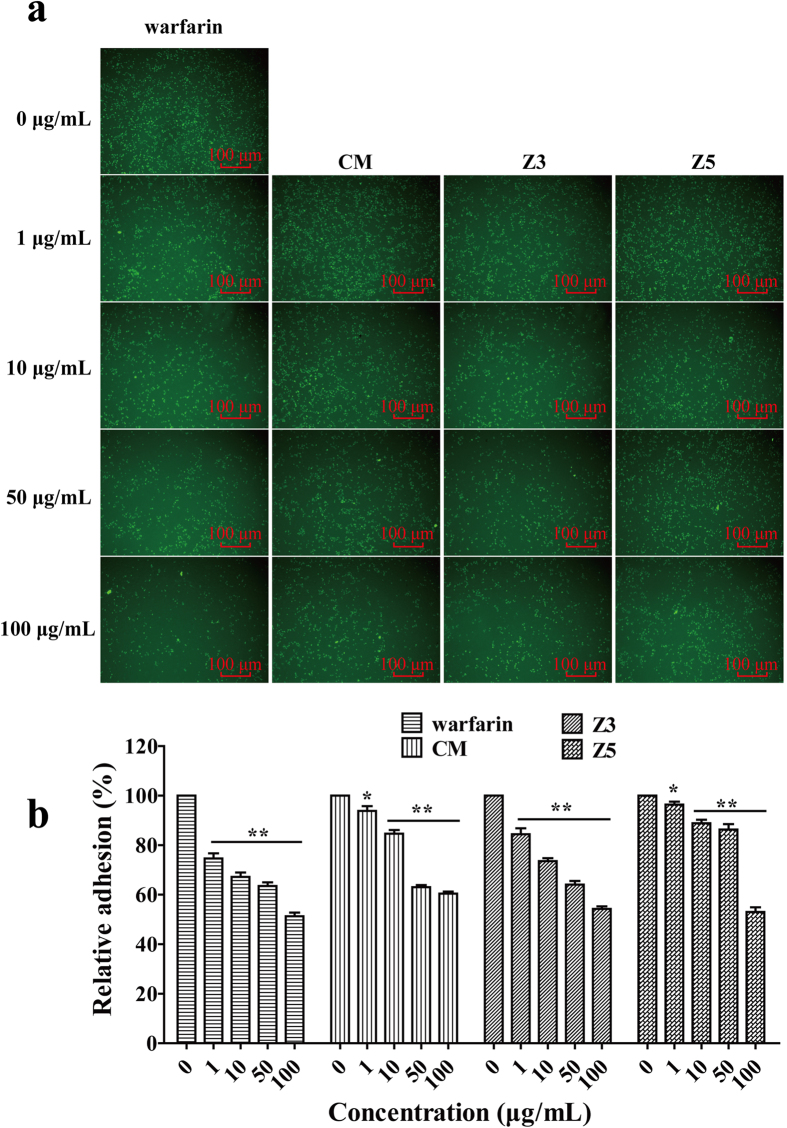
Concentration-dependent inhibition by warfarin, Z3, Z5 and CM on adhesion of HCT116 to HUVECs. (**a)** warfarin, CM, Z3 and Z5 interfered with adhesion of Rhodamine 123-labeled HCT116 cells to HUVEC monolayer stimulated by IL-1β (1 ng/mL). (**b**) quantitative analysis of the concentration-dependent effect of warfarin, CM, Z3, and Z5 and warfarin on adhesion of HCT116 cells to HUVECs. Bars represent the mean ± SEM (n = 3). *P < 0.05, and **P < 0.01, compared with the control.

We chose human umbilical vein endothelium cells (HUVECs) and HCT116 to simulate the adhesion of CTCs to vascular intima. Warfarin was used as a positive control because of drug structure-efficacy relationship. The experiment showed that the number of Rhodamine 123-labeled HCT116 cells adhered to HUVECs was gradually decreased with the increasing concentrations of warfarin, CM, Z3 and Z5 up to 100 μg/mL (Fig. 4a). The 50% of inhibition on the adhesion of HCT116 cells to HUVECs could be reached by warfarin, CM, Z3 and Z5 around 100 μg/mL (Fig. 4b), and the concentration was well below the IC_50_ level, suggesting that these molecules may have a specific effect on the cell-cell adhesion through the mechanism of down-regulating EpCAM expression (Fig. 3b,c).

### Western blot analysis of cellular EpCAM expression regulated by CM, Z3 and Z5

**Figure 5:**
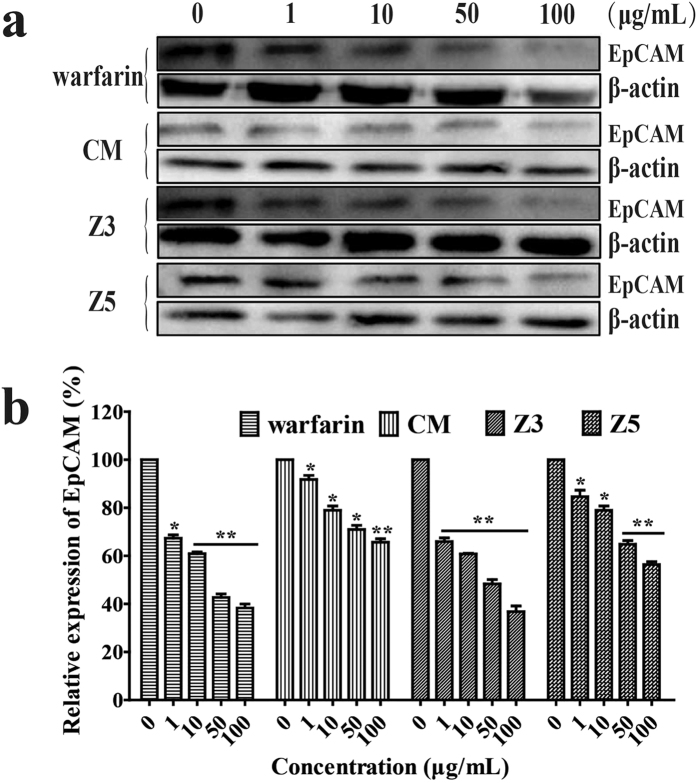
Western blot analysis of EpCAM expression on HCT116 cells in the presence of warfarin, Z3, Z5 and CM. (**a)** Western blot analysis of EpCAM expression on HCT116 cells pretreated with warfarin, Z3, Z5 and CM (0, 1, 10, 50 and 100 μg/mL) for 24 h. Band intensity was quantified by using Image Lab analysis software. (**b**) quantitative analysis of the Western blot assay demonstrated the concentration-dependent inhibition of warfarin, Z3, Z5 and CM on expression of EpCAM on HCT116 cells. Z3 inhibited the expression significantly. Bars represent the mean ± SEM (n = 3). *P < 0.05, and **P < 0.01, compared with the control.

To verify the role of CM, Z3 and Z5 in down-regulating expression of EpCAM observed by using flow cytometry (Fig. 3), we further conducted the western blot assay to examine if these compounds could inhibit EpCAM expression at protein level^23^. Warfarin and Z3 significantly down-regulated EpCAM expression after 24-h incubation of the two compounds with HCT116 cells. Next are Z5 and CM, both showed significant down-regulation of EpCAM expression in a concentration-dependent manner. The results were shown in the real western blot images (Fig. 5a) and quantitative analysis (Fig. 5b).

### Comparison in anticoagulation *in vivo* between warfarin and Z3

**Figure 6:**
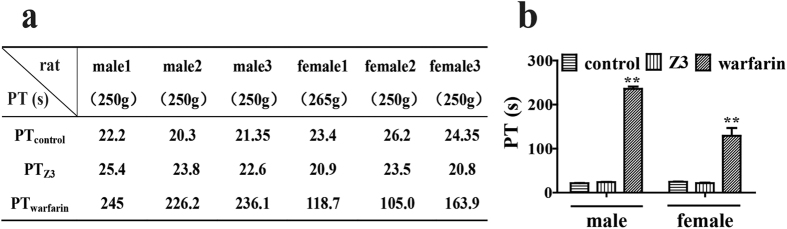
Comparison in prothrombin time between Z3 and warfarin. (**a)** changes of prothrombin time (PT; second) in rats orally administered with Z3 or warfarin (0.5 mg/kg/day for 5 days). The individual PT was significantly prolonged by oral warfarin, but not by oral Z3. (**b)** Quantitative analysis showed a significant difference in rat PT between warfarin and Z3; **P < 0.01, compared with the control.

The anticoagulant effect of warfarin results from its *in vivo* inhibition of synthesis of four vitamin K-dependent clotting factors and degradation of these existing clotting factors^24^. To compare the efficacy in terms of anticoagulation between warfarin and Z3, we selected rats as a model to investigate the *in vivo* similarities and differences in anticoagulation between the two compounds. After 5 days of oral administration of the two compounds to the rats (0.5 mg/kg/day), the rat prothrombin time was significantly prolonged by warfarin in both males and females. However, Z3 did not significantly affect the prothrombin time in comparison with the untreated control (Fig. 6a,b). The result suggests the newly-explored coumarin-like compounds may have a safety profile better that warfarin when used as the EpCAM-based anti-adhesion therapy for cancer metastatic prevention.

## Discussion

The present study reported, for the first time, that the coumarin-like compounds (Z3-Z5) extracted from *M. paniculata* could specifically inhibit epithelial cell adhesion molecule, namely EpCAM, without affecting cancer cell viability. We demonstrated that these compounds down-regulated EpCAM expression on the cell surface by using flow cytometry (Fig. 3). We further showed that Z3 and Z5 inhibited cellular expression of EpCAM by running western blotting (Fig. 5). The inhibition by the coumarin-like compounds of EpCAM expression was first reported, and this discovery may further explain the anti-coagulation mechanism of warfarin by which it could directly inhibit hetero cellular adhesion as we observed in the *in vitro* assay. This result may, in part, contribute to warfarin’s anti-coagulation *in vivo*, which has not been reported so far. Current understanding of warfarin’s anticoagulant effect is limited to its inhibition of synthesis of four vitamin K-dependent clotting factors and its degradation of these existing factors. It usually takes approximately 3–5 days for the existing factors to be degraded after administration of warfarin, which is why we designed the experimental period for 5 days (Fig. 6).

Many TCMs are used as the raw extract^19^, but single component may have its specific effect differently from each other. Therefore, we tried to separate single components from the extract of *M. paniculata*. to analyze their individual effects. Five components were obtained and characterized. They are murpanidin (C_15_H_16_O_5_, Z1), isomexoticin (C_16_H_20_O_6_, Z2), phebalosin (C_15_H_14_O_4_, Z3), murpanicin (C_17_H_20_O_5_, Z4), and murralongin (C_15_H_14_O_4_, Z5), respectively. They all showed very low cytotoxic activity (Fig. 3a). However, they regulated the EpCAM expression differently. Z1 and Z2 upregulated the expression, whereas, Z3, Z4 and Z5 did oppositely. We then used the extract mixture or its resembling component mixture (which was reconstituted by adding each component together according to their original % proportion in the extract), in the bioassays to compare the effect of CM with that of the individual components. CM produced a collective effect on EpCAM expression similar to what Z3 exhibited in EpCAM expression and cell-cell adhesion. Therefore, we concluded that a botanical raw extract, although it contains different agonistic and antagonistic components (or Ying and Yang components in the phrase of TCM), will exhibit a comprehensive pharmacological effect after harmonizing each individual effects, and only the leading effect will be represented. The conclusion was demonstrated in the present *in vitro* bioassays (Figs 3, 4, 5).

The present study found that there was a significant difference in prothrombin time between rat warfarin and Z3 groups. The result clearly indicates that although Z3 contains the same coumarin main structure as warfarin does, the difference in chemical structure, especially in side-chain between the two compounds decides the difference in their pharmacological effects. First, warfarin exhibits a potent anti-coagulative effect *in vivo*, and is clinically the first choice for anti-coagulation^25^. Whereas, the tested coumarin-like compounds, namely, Z3, Z5, and CM, may be a good candidate for inhibiting adhesion of cancer cells to vascular intima because they specifically target at cell-cell adhesion at low concentrations without significant cytotoxicity and anti-coagulation.

Completely different from the traditional anti-cancer drug development that aims at finding cytotoxic agents to kill cancer cells at IC_50_ as low as nM levels, our strategy focuses on interfering with the starting point of the CTC activation-adhesion metastasis cascade with the hypothesis that if the CTCs fail to adhere the endothelial layer of distant metastatic tissues, they may die due to the loss of matrix-derived survival signals, circulatory shear stress, and/or anoikis^26^. Following this idea, we found many safe and effective cancer metastatic chemopreventives, and demonstrated their efficacy *in vitro* and *in vivo*^3^,^9^,^19^,^27^. It is our hope that this paradigm-shafting idea and discovery could open a new avenue to develop products to serve well for the asymptomatic cancer survivors for preventing future cancer metastasis after primary treatment.

## Methods

### Reagents

Human interleukin-1 beta (IL-1*β*) was purchased from Cell Signaling Technology Inc. Mouse anti-human CD29 (Integrin *β*1)-PE, mouse anti-human CD44-FITC, mouse anti-human CD49c and CD49e (Integrin *α*3 and Integrin *α*5)-PE, anti-Human CD326 (EpCAM)-PE and PE mouse IgG1 kappa isotype control antibodies were all obtained from Becton Dickinson (BD) Pharmingen.

### Rats

SD (Sprague–Dawley) rats were obtained from the LRC laboratory animal (Shanghai, China). They were housed in a separate room, and caged according to sex and dose levels. They were housed in specific pathogen-free conditions. They were kept at 25 ± 2 °C under the conditions of 12 h/12 h light/dark cycle, 50–60% relative humidity and received water and food ad libitum. All animal experiments were done according to the protocols approved by the Animal Research Committee of Fuzhou University. Animal welfare and experimental procedures were performed strictly in accordance with the institutional care and use of laboratory animal guidance^28^.

### Extraction procedures

The shade-dried roots of *Murraya paniculata* (15 g) were powdered, followed by extraction with 450 mL of 80% acidic ethanol (pH 3) under refluxing for 3 times (5 h for each time). The remains were re-suspended with water and then filtrated. The pH of the filtrate was adjusted to 9 with aqueous ammonia, and the filtrate was then extracted with ethyl acetate for five times. The ethyl acetate layer was dried by rotary evaporation, giving the extract of coumarin (CM).

### HPLC analysis and separations of CM

The analytic and semi-preparative HPLC was performed on an Waters HPLC system containing 2695 separation module and 2489 UV-Vis detector. The analytic HPLC method was similar to what we reported previously^29^. A 20 μL aliquot of CM was auto-injected and chromatographed on an Waters Sunfire C18 column (15 cm × 4.6 mm, 5 μm) maintained at 35 °C with isocratic elution of methanol-water (45: 55, v:- v) at a flow rate of 1.0 mL/min. The UV-Vis detection wavelength was set at 310 nm. Semi-preparative HPLC was performed on an Agela Venusil XBP C18 column (25 cm × 10 mm, 5 μm) maintained at 35 °C. The elution program was similar to that in the analytic HPLC method except for a larger injection volume (200 μL) and a faster flow rate (3.0 mL/min). Five components (Z1, Z3, Z4, Z5, Z2) were collected successively corresponding to the HPLC peak shown on Fig. 2b, which were then dried by rotary evaporation and lyophilizated to give amorphous powders, respectively.

### Spectroscopic characterization

UV–Vis spectrum of CM in methol was collected by PE Lambda-750 UV/VIS/NIR spectrophotometer. Quartz cells with path length of 1 cm were used for detection. Mass spectra were analyzed by Exactive Plus Orbitrap LC-MS (Thermo Fisher Scientific). NMR analysis was carried out with an AVANCE III 500 MHz NMR system (Bruker, Switzerland), with deuterated dimethyl sulphoxide (DMSO) as the solvent.

### Cell cultures

Human colon cancer HCT116 cells were obtained from the Cell Bank of Type Culture Collection of Chinese Academy of Sciences. The cells were cultured in McCoy’s 5A medium supplemented with 10% fetal bovine serum, 100 units/mL penicillin and 100 mg/mL streptomycin. Human umbilical vein endothelial cells (HUVECs) were separated and cultured as we described previously^9^. HUVECs were maintained in 1% gelatin-coated tissue culture flasks in M199 (Gibco) medium supplemented with 20% FBS, 8 units/mL heparin, 100 mg/mL ECGS, 100 units/mL penicillin and 100 mg/mL streptomycin and were discarded after 6 passages. The cells were all maintained at 37 °C and 5% CO_2_ in a humidified atmosphere.

### Cytotoxicity assay

Cell viability was assessed using 3-(4,5-dimethylthiazol-2-yl)-2,5-diphenyltetrazolium bromide (MTT) assay as we described previously^9^,^30^. In short, 100 μL 1 × 10^4^ per well HCT116 cells were cultivated in 96-well plate with McCoy’s 5A medium for 24 h and then treated with various concentrations of warfarin, CM, Z1~Z5 (1–1000 μg/mL) for 24 h. Finally, MTT solution (5 mg/mL) was added, and the cells were incubated for another 4 h in the medium without phenol red and serum. The MTT-formazan formed by metabolically viable cells was dissolved in 100 μL DMSO and shaken for 10 min. The OD_570 nm_ was recorded by ELISA reader. The inhibition of treated cells was calculated as follows:


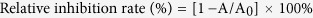


A: OD value of the experimental group; A_0_: OD value of the parallel solvent control group.

### Flow cytometry

Flow cytometric analysis of CAMs expression on cells was performed on a BD FACSAriaIII cell sorter with laser excitation set at 488 and 633 nm, as we described previously^9^. BD FACSDiva software provided with the system was employed for data acquisition and initial data analysis. Forward versus side scatter histograms were utilized to gate on single intact cells. The data were collected in FCS format with the subsequent analysis based on 1 × 10^4^ cells to meet the light scatter criteria. PE and PI dyes were excited by 488 nm laser and detected through 585 and 530 nm bandpass filters. APC dye was excited by 633 nm laser, and the fluorescence emission was detected through 660 nm bandpass filter. FITC dye was excited by 488 nm and detected through 530 nm bandpass filter. In our study, the inhibition effect of warfarin, CM, Z1~Z5 on the expression of adhesion molecules of HCT116 were estimated by flow cytometry for investigating the possible mechanism. HCT116 cells were cultivated on 6-well culture plates followed by the treatment of warfarin, CM, Z1~Z5 (1–100 μg/mL) for 24 h. The cells and primary antibodies were incubated at 4 °C for 30 min protected from light. After washing with staining buffer, the cell suspension was passed through the flow cell of the FACSAriaIII flow cytometer for analysis. The data were processed by FlowJo software and expressed as the mean fluorescent intensities.

### Adhesion assay of HCT116 to HUVECs

Fluorescence microscope photographed method was used to quantify the adhesion of HCT116 cancer cell adhesion to endothelial cells, as we described previously^3^,^9^. HUVECs were seeded in a 24-well plate and cultured to 90% confluence, followed by stimulation with 500 μL of 1 ng/mL IL-1*β* for 4 h. HCT116 cells labeled with Rhodamine 6G were suspended in McCOY’s 5 A media with different concentrations of warfarin, CM, Z3, Z5, and seeded to the wells covered with HUVECs. After another 1 h of incubation, the nonadhered cells were gently washed off with PBS for twice. The adhered HCT116 cells were photographed by an inverted fluorescence microscope (Zeiss, Germany) and counted for number. The mean inhibition of adhesion for 10 random visual fields was calculated by the equation:


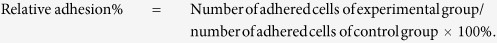


### Western Blot analysis

5 × 10^5^ per well HCT116 cells were seeded on 6-well tissue culture plates and incubated in McCOY’s 5A media for 24 h, and then treated with various concentrations of warfarin, CM, Z3 and Z5 (1–100 μg/mL) for 24 h. The cell extracts were prepared by lysis in RIPA on ice. Then equal amounts of protein were separated by sodium dodecylsulfate-polyacrylamide gel electrophoresis, and transferred to polyvinylidene difluoride (PVDF, Bio-Rad) membranes. The membranes were blocked in 5% skim milk for 1 h and probed with the corresponding primary antibodies overnight at 4 °C. After washing, the membranes were incubated with the following secondary antibodies for 1 h at room temperature. Chemiluminescent signals were generated using a Super Signal West Pico Chemiluminescent Substrate kit (Pierce), and detected by the ChemiDoc XRS system (Bio-Rad). Band intensity was quantified with Image Lab analysis software (Bio-Rad). Total EpCAM expression was normalized to the levels of loading control β-actin.

### Anticoagulation experiment analysis

The rats (weighed c.a. 250 g) were randomly divided into three groups, 3 male and 3 female each group. The drugs for test, Phebalosin (Z3) and warfarin, were dissolved in 1% ethanol solution at the concentration of 0.5 mg/mL. A dosage of 0.5 mg/Kg for each drug was orally administrated every day for 5 days. The group administrated with 1% ethanol aqueous was set as the control. On the sixth day, the blood was drawn from the rats’ tail vein, and mixed well with sodium citrate solution (3.8%) in 9: 1 volume ratio. The blood samples were centrifuged at 1000 rcf for 15 min and the upper plasma were taken for Prothrombin Time (PT) tests by a coagulation instrument. Each sample was tested for three times.

### Statistical analysis

SPSS statistics software was used to analyze the Data in our study. Statistical analysis was performed using the Student’s test. A *P*-value < 0.05 was considered statistically significant, and *P* < 0.01 to be extremely statistically significant.

## Additional Information

**How to cite this article**: Shao, J. *et al.* Warfarin and coumarin-like *Murraya paniculata* extract down-regulate EpCAM-mediated cell adhesion: individual components versus mixture for studying botanical metastatic chemopreventives. *Sci. Rep.* **6**, 30549; doi: 10.1038/srep30549 (2016).

## Supplementary Material

Supplementary Information

## Figures and Tables

**Figure 1:**
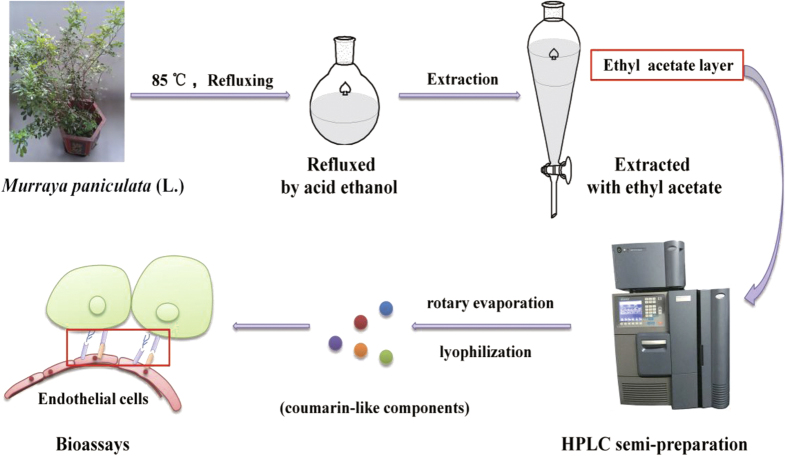
Schematic of bioactivity-guided fast screen for cancer metastatic chemopreventive materials from raw extracts of *Murraya paniculata.* Procedures of extracting active components from the plant roots: the roots were collected, dried, and powdered. The raw powder was refluxed with 80% acid ethanol and the concentrated residual was extracted with ethyl acetate. The extract was subjected to HPLC semi-preparation column separation, which resulted in five coumarin-like components in amorphous powders after rotary evaporation and lyophilization. The powders were tested for their pharmacological activities.
