# Supplementary material for: Warfarin and coumarin-like Murraya paniculata extract down-regulate EpCAM-mediated cell adhesion: individual components versus mixture for studying botanical metastatic chemopreventives
Source: Sci Rep. 2016 Aug 2;6:30549. doi: 10.1038/srep30549 (PMC4969747; doi:10.1038/srep30549)
Supplement: Supplementary Information [file srep30549-s1.pdf]

Supporting Information

Warfarin and coumarin-like *Murraya paniculata* extract down-regulate EpCAM-mediated cell adhesion: individual components versus mixture for studying botanical metastatic chemopreventives

Jingwei Shao [1,2,#], Suxia Zhou [1,#], Zhou Jiang [1,2,#], Ting Chi [1], Ji Ma [1]. Minliang Kuo [3], Alan Yueh-Luen Lee [4], Lee Jia [1,2,*]

[1] Cancer Metastasis Alert and Prevention Center, and Biopharmaceutical Photocatalysis, State Key Laboratory of Photocatalysis on Energy and Environment, Fuzhou University, Fuzhou 350002, China.

[2] Fujian Provincial Key Laboratory of Cancer Metastasis Chemoprevention, Fuzhou University, Fuzhou, China

[3] Graduate Institute of Biomedical Sciences, College of Life Science, National Taiwan University, Taipei, Taiwan

[4] National Institute of Cancer Research, 35 Keyan Road, Zhunan, Miaoli35053, Taiwan

* Corresponding author: Lee Jia. Address: Cancer Metastasis Alert and Prevention Center, 3rd floor, Science Building, 523 Industry Road, Fuzhou, Fujian, 350002, China.

Phone: 086-1515-963-0201. Email: pharmlink@gmail.com; or cmapcjia1234@163.com

# These authors contributed equally to this work.

Supplementary Table S1. ^{13}C-NMR spectral data for coumarin derivatives(500MHz, DMSO-d_6, δ, ppm)

Compounds	Murpanidin(Z1)	Isomexoticin(Z2)	Phebalosin(Z3)	Murpanicin(Z4)	Murralongin(Z5)
Formula	$C_{15}H_{16}O_5$	$C_{16}H_{20}O_6$	$C_{15}H_{14}O_4$	$C_{17}H_{20}O_5$	$C_{15}H_{14}O_4$
Structure	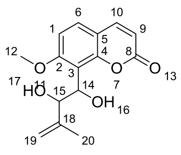	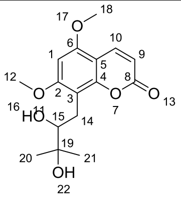	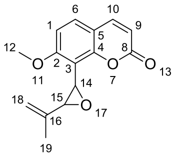	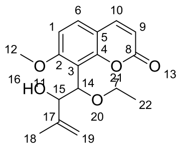	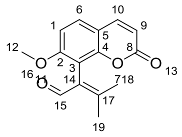
Position	δc	Δc	Δc	δc	δc
1	109.0	92.06	109.0	108.9	108.66
2	160.5	161.01	160.66	160.5	160.67
3	117.7	108.56	112.74	113.1	117.66
4	153.1	155.51	154.25	153.5	152.48
5	112.6	103.18	115.64	112.7	112.27
6	129.2	154.17	129.41	129.8	129.22
8	160.8	162.03	162.1	161.2	160.27
9	113.1	110.58	115.6	114.9	112.59
10	145.3	139.46	145.3	145.3	145.17
12	56.7	56.74	56.49	56.85	56.70
14	68.2	26.05	57.00	76.55	129.65
15	77.3	76.83	74.47	76.39	189.98
16			147.7		
17				145.76	145.17
18	146.2	56.48	112.74	17.19	24.91
19	112.2	72.39	19.03	112.58	19.87
20	17.48	25.09			
21		25.09		65.11	
22				15.71	

Supplementary Table S2. ^{1}H-NMR spectral data for coumarin derivatives (125MHz, DMSO-d_6, δ, ppm)

Compounds	Murpanidin(Z1)	Isomexoticin(Z2)	Phebalosin(Z3)	Murpanicin(Z4)	Murralongin(Z5)
Formula	$C_{15}H_{16}O_5$	$C_{16}H_{20}O_6$	$C_{15}H_{14}O_4$	$C_{17}H_{20}O_5$	$C_{15}H_{14}O_4$
Structure	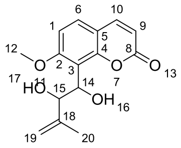	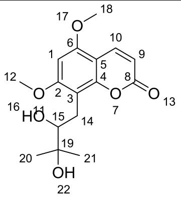	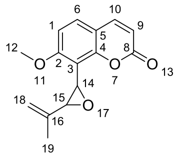	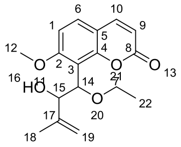	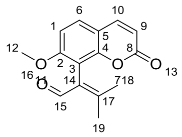
Position	δH	δH	Δh	δH	δH
1	7.05	6.65	7.07	7.07	7.14
6	7.58		7.60	7.60	7.71
9	6.28	6.15	6.28	6.28	6.28
10	7.96	8.00	7.97	7.97	8.04
12	3.88(3)	3.95(3)	3.88(3)	3.88(3)	3.88 (3)
14	5.00	2.77(2)	5.07	4.87	
15	4.70	3.46	4.91	4.93	10.19
16	5.12	4.11		5.07	
17	5.00				
18		3.95(3)		1.48(3)	1.71(3)
19	4.42,4.50		4.39,4.51	4.39,4.51	2.41(3)
20	1.53(3)	1.13(3)	1.48(3)		
21		1.13(3)		3.43(2)	
22		1.25		1.06(3)	
